# Heterogene neuropsychiatrische Phänotypen bei zwei erwachsenen Patient*innen mit 22q11.2-Deletionssyndrom (DiGeorge-Syndrom): ein Fall für RDoC?

**DOI:** 10.1007/s00115-021-01226-6

**Published:** 2021-11-04

**Authors:** Peter Praus, Urs Braun, Melanie Bleich, Andreas Meyer-Lindenberg, Oliver Hennig

**Affiliations:** 1 Klinik für Psychiatrie und Psychotherapie, Zentralinstitut für Seelische Gesundheit, J5, 68159 Mannheim, Deutschland; 2 Klinik für Forensische Psychiatrie, Pfalzklinikum für Neurologie und Psychiatrie, Weinstraße 100, 76889 Klingenmünster, Deutschland

**Keywords:** Mikrodeletionssyndrome, DiGeorge-Syndrom, ADHS, Autismus-Spektrum-Störung, RDoC, Microdeletion syndromes, DiGeorge’s Syndrome, ADHD, Autism spectrum disorder, RDoC

## Abstract

Das DiGeorge-Syndrom ist eines der häufigsten Mikrodeletionssyndrome und bedingt ein erhöhtes Risiko für neuropsychiatrische Störungen der Intelligenz, der sozialen Kommunikation und der Exekutivfunktionen sowie psychotische Störungen. Im Falle des vorgestellten männlichen Patienten handelt es sich um die seltene Beschreibung eines Tourette-Syndroms auf der Grundlage eines 22q11.2-Mikrodeletionssyndroms. Die folgenden zwei Fallbeispiele demonstrieren die Vielfalt assoziierter klinischer Präsentationen, selbst auf der Grundlage einer übereinstimmenden und umschriebenen genetischen Aberration. Eine Charakterisierung solcher Patient*innen im Kontext der klinisch-wissenschaftlichen Praxis anhand der Research Domain Criteria (RDoC) ermöglicht eine transdiagnostische Beschreibung der überlappenden wie auch spezifischen neuropsychiatrischen Funktionseinschränkungen. Eine solche dimensionale Charakterisierung erlaubt somit potenziell auch eine genauere Differenzierung pleiotroper Assoziationen zwischen Genotyp und Phänotyp.

## Abstract (en)

DiGeorge’s syndrome is one of the most frequent microdeletion syndromes and is associated with a high risk for neuropsychiatric disorders of intelligence, social communication and executive functioning as well as psychotic disorders. The male patient described here represents one of the rare descriptions of Tourette’s syndrome on the basis of a 22q11.2 microdeletion syndrome. The following two case studies demonstrate the variety of related clinical presentations. A characterization of these patients in a clinical and scientific context by the means of Research Domain Criteria (RDoC) enables a transdiagnostic description of overlapping as well as specific neuropsychiatric functional impairments. Possibly, this dimensional characterization might also facilitate a more exact differentiation of pleiotropic associations between genotype and phenotype.

## Hintergrund

Das 22q11.2-Deletionssyndrom, auch als DiGeorge-Syndrom bekannt, beruht auf einer hemizygoten Mikrodeletion von 1,5 bis 3 Megabasen auf dem langen Arm von Chromosom 22 und tritt mit einer Prävalenz von 1:3000 bis 1:6000 Lebendgeburten auf [10]. Somit gehört es zu den häufigsten chromosomalen Mikrodeletionssydromen überhaupt [14]. Die Rolle der betroffenen Gene bei der Entstehung neuropsychiatrischer Manifestationen wird zunehmend besser verstanden. Sie sind höchstwahrscheinlich auch das Ergebnis einer komplexen Interaktion von Genen untereinander im Zusammenspiel mit Umweltfaktoren [9]. Die Prävalenz von Autismusspektrumstörungen bei Individuen mit 22q11.2-Deletionssyndrom wird mit ca. 10–50 % angegeben [20]. Auch anhand von Registerstudien konnte ein signifikant erhöhtes Risiko von Betroffenen nachgewiesen werden, eine Autismusspektrumstörung, eine hyperkinetische Störung, eine Intelligenzminderung oder eine Schizophreniespektrumstörung bzw. irgendeine psychische Störung zu entwickeln [8]. In der Literatur finden sich lediglich zwei Beschreibungen eines Tourette-Syndroms auf dem Boden eines 22q11.2-Deletionssyndroms [6, 19]. Seit ihrer Einführung im Jahr 2012 bieten die Research Domain Criteria ein leistungsstarkes Rahmenwerk für die Operationalisierung und Unterteilung komplexer psychischer Störungen in einer Weise, die sowohl neurobiologisch orientierte als auch klinische Ansätze integriert (https://www.nimh.nih.gov/research/research-funded-by-nimh/rdoc/index.shtml). Die biologische Komplexität kategorial definierter Erkrankungen führt zu ausgeprägten pleiotropen Assoziationen sowohl häufiger als auch seltener genetischer Varianten mit diesen Phänotypen. Die 22q11-Mikrodeletion ist hier keine Ausnahme. Eine Charakterisierung dieser Individuen jenseits kategorialer diagnostischer Kriterien anhand von RDoC könnte exemplarisch zur präzisen mehrdimensionalen neurobiologisch fundierten Diagnostik beitragen.

## Fallbeispiele

Die beiden hier vorgestellten Patientin*innen stellten sich in den Spezialambulanzen für hochfunktionalen Autismus bzw. ADHS im Erwachsenenalter am Zentralinstitut für Seelische Gesundheit zur Diagnostik und Beratung vor. Beide Patient*innen erklärten ihr schriftliches Einverständnis mit der Veröffentlichung des jeweiligen Falles zu wissenschaftlichen Zwecken in anonymer Form.

### Patientin A

#### Eigen- und Fremdanamnese.

Die zum damaligen Zeitpunkt 38-jährige Patientin stellte sich im Mai 2018 in der Spezialambulanz für Autismus vor. Sie berichtete von Reizüberflutungserleben in unterschiedlichen Situationen. Ferner imponierten Einschränkungen der Blicksteuerung und ausgeprägte Defizite der sozialen Kommunikation, die auf eine mangelnde Fähigkeit, Emotionen und soziale Konstrukte zu erkennen und zu verstehen, sowie ein wörtliches Sprachverständnis zurückgeführt wurden. Es wurde ein ausgeprägtes Bedürfnis nach Routinen, eine intensive Beschäftigung mit einer überschaubaren Anzahl nahe verwandter Themen sowie eine Überempfindlichkeit gegenüber leichten Berührungen und Geräuschen beschrieben. Die Patientin gab an, regelmäßig in einem Betrieb in gemeinnütziger Trägerschaft zu arbeiten; sie lebe in einer ambulant betreuten Wohngemeinschaft. Eine ältere Schwester sowie eine Tante bestätigten die Angaben der Patientin. Zudem berichteten die Angehörigen von stereotypen Bewegungsmustern des Oberkörpers, der Arme sowie der Hände im Zusammenhang mit Stress, einer motorischen Ungeschicklichkeit sowie einem erheblich reduzierten Orientierungsvermögen in ungewohnten Umgebungen.

#### Psychiatrische und Aktenanamnese.

Den vorgelegten Grundschulzeugnissen waren ein zurückhaltendes Verhalten mit geringer Beteiligung am Unterricht, Störungen der Aufmerksamkeit, mangelnde Sorgfalt und Genauigkeit bei der Erledigung von Aufgaben sowie Schwierigkeiten bei veränderten Aufgabenstellungen und der korrekten Umsetzung von Arbeitsanweisungen zu entnehmen. Den vorgelegten ärztlichen Befundberichten über stationäre und teilstationäre psychiatrische Behandlungen im Zeitraum von 2006 bis 2018 waren in unterschiedlichen Kombinationen die folgenden Diagnosen zu entnehmen: rezidivierende depressive Störung, Panikstörung, Borderline-Störung, posttraumatische Belastungsstörung, dissoziative Störung, artifizielle Störung, unterdurchschnittliche Intelligenz und Intelligenzminderung.

#### Somatische Anamnese.

Diesbezüglich war ein 22q11.2-Mikrodeletionssyndrom mit konsekutivem Immundefekt im Sinne einer Leukopenie, einer Leukämieerkrankung im Jahr 1997 sowie einer Tuberkuloseerkrankung im Jahr 1982 zu konstatieren. 2014 erfolgte aufgrund der Angabe von Krampfanfällen eine ausführliche Epilepsiediagnostik; das EEG und alle weiteren Untersuchungen, u. a. eine kranielle MRT, erbrachten keinen richtungsweisenden Befund. Eine antiepileptische Pharmakotherapie erfolgte nie.

#### Befunde.

Psychopathologisch fielen ein eingeengter, verarmt und stockend wirkender formaler Gedankengang mit Hang zu konkretistischem Denken, ein nivellierter Affekt mit reduzierter Resonanzfähigkeit, eine kaum modulierte Mimik und Gestik in Kombination mit einer monoton wirkenden Sprachprosodie sowie intermittierende motorische Stereotypien im Sinne „wippender“ Bewegungen des Oberkörpers im Sitzen auf. Der restliche psychopathologische Befund war unauffällig. Im neurologischen Untersuchungsbefund ergaben sich außer einem etwas vergröbert wirkenden Gangbild, diskreten kraniofazialen Dysmorphiezeichen im Sinne einer schmalen Mundpartie, abstehender, dysplastischer Ohren sowie einer prominenten, langgesteckten Nasenpartie mit leicht hypoplastisch anmutenden Nares sowie einer relativ geringen Körpergröße von 156 cm keine anderweitigen auffälligen Befunde.

In den verwendeten deutschen Versionen der Screeninginstrumente (AQ [3]; EQ [1], WURS‑k [18]; FSK [5], SRS [4] und MBAS [11]) zeigten sich durchgehend auffällige Werte. Im Autism Diagnostic Observation Schedule (ADOS‑2; [12]) erreichte die Patientin einen Punktwert von 11, passend zu einer Autismusspektrumstörung. Im Reading the Mind in the Eyes Test (RMET; [2]) wurden 8 Punkte erzielt, was auf signifikante Defizite der Emotionserkennung hinweist.

Eine sprachfreie Intelligenztestung (CFT-20‑R, Standard Progressive Matrices) erbrachte Gesamt-IQ-Werte von 77 bzw. 61. Das verbale Intelligenzniveau (MWT-B) lag bei 85 IQ-Punkten. Die kognitive Verarbeitungsgeschwindigkeit sowie die geteilte Aufmerksamkeit (TMT‑A, TMT-B) waren durchschnittlich. Im Farb-Wort-Interferenz-Test (FWIT) war keine Interferenzneigung nachweisbar. In einer Untersuchung des Arbeitsgedächtnisses (WMS-R) erzielte die Patientin im Vergleich mit ihrer Altersgruppe knapp durchschnittliche Werte.

#### Einschätzung.

Passend zu bisherigen Erkenntnissen zu Entwicklungsstörungen bei Individuen mit 22q11.2-Deletionssyndrom [20] zeigte sich bei der Patientin ein noch durchschnittliches bis leicht unterdurchschnittliches kognitives Leistungsniveau, ohne dass klinische Kriterien einer Intelligenzminderung erfüllt waren. Zwar können „Theory-of-mind“-Defizite bei Individuen mit 22q11.2-Deletionssyndrom unabhängig vom Vorliegen einer Autismusspektrumstörung vorkommen [13]; doch lagen im Falle der Patientin zusätzlich Einschränkungen der Sprachpragmatik und des Verständnisses für soziale Konstrukte sowie eine Fixierung auf ein stereotypes Repertoire repetitiver Handlungsmuster in Kombination mit motorischen Stereotypien und sensuellen Überempfindlichkeiten vor. Daher konnte die kategoriale Diagnose einer Autismusspektrumstörung bestätigt werden. Passend hierzu zeigten sich insbesondere Auffälligkeiten in den Subkonstrukten „soziale Kommunikation“ und „Wahrnehmung und Verständnis – Andere“ der RDoC-Domäne „soziale Prozesse“. Die Charakteristika der Motorik konnten mithilfe der RDoC-Domäne „sensomotorische Systeme“ adäquat abgebildet werden.

### Patient B

#### Eigen- und Fremdanamnese.

Die Vorstellung des 20-jährigen Patienten erfolgte im November 2019 vor dem Hintergrund einer Aufmerksamkeitsdefizit-/Hyperaktivitätsstörung (ADHS) in Kombination mit einem Tourette-Syndrom aufgrund eines Beratungswunsches hinsichtlich medikamentöser Therapieoptionen. Eine Behandlung mit Tetrahydrocannabinol hatte zuletzt keine Besserung der Tic-Symptomatik erbracht, jedoch zu einer Zunahme der Konzentrationsstörung geführt. Aktuell wurden motorische Tics in Form von Blinzeln und Grimassieren, Stimmungsschwankungen, motorische und innere Unruhe sowie gesteigerte Impulsivität beklagt. Ferner wurde eine anhaltende depressive Verstimmung in Kombination mit aggressiven Zwangsgedanken und Zwangshandlungen angegeben. Nach dem Besuch einer Förderschule sei er nun in einer Werkstatt für behinderte Menschen tätig; er lebe mit den Eltern und einer jüngeren Schwester in einem gemeinsamen Haushalt.

#### Psychiatrische und Aktenanamnese.

Es lag eine umfangreiche Akte der Klinik für Psychiatrie und Psychotherapie des Kindes- und Jugendalters am Zentralinstitut für Seelische Gesundheit aus dem Zeitraum März 2012 bis Mai 2013 vor. Nach unauffälliger Geburt seien in den ersten Lebensjahren eine Gedeihstörung und ein verzögertes Längenwachstum aufgefallen. 2009 sei die erste psychiatrische Behandlung im Zusammenhang mit einer kombinierten komplexen Tic- und ADHS-Symptomatik erfolgt. Im Verlauf erfolgten diverse medikamentöse Therapien mit Methylphenidat und Atomoxetin einerseits und andererseits Tiaprid, Risperidon, Clonidin, Pimozid und Aripiprazol. Unter einer Kombinationstherapie mit Aripiprazol und Pimozid sei eine stabile Teilremission der Tic-Störung beobachtet worden.

Ferner seien auch Schwierigkeiten der sozialen Interaktion und regressive Verhaltensweisen aufgefallen; pädagogische Anleitung und Übung hätten hier keine Besserung ergeben. Infolge einer ergotherapeutischen Behandlung seien hingegen eine emotionale Stabilisierung und eine Verbesserung des Selbstkonzeptes beobachtet worden.

Eine ambulante differenzierte Intelligenzdiagnostik im Jahr 2018 (WISC-V) hatte zuletzt unterdurchschnittliche allgemeine Lern- und Leistungsmöglichkeiten bestätigt.

#### Somatische Anamnese.

Es lag eine heterozygote Mikrodeletion der chromosomalen Bereiche 22q11.21–q11.23 vor, des Weiteren ein Hypopituitarismus mit konsekutivem Minderwuchs und Hormonbehandlung an einem universitären Zentrum in der Vergangenheit. Darüber hinaus wurde eine habituelle Luxation der linken Patella angegeben.

#### Befunde.

Psychopathologisch imponierten ein hyperkinetisches Syndrom mit Stimmungsschwankungen bei gesteigerter Impulsivität und Irritabilität, kombinierte komplexe vokale und motorische Tics sowie Zwangsgedanken und -handlungen. In der Yale Globale Tic-Schweregrad-Skala (YGTSS) zeigte sich eine schwere Gesamtbeeinträchtigung.

Im körperlichen Untersuchungsbefund waren ein Minderwuchs und Dysmorphiezeichen in Form einer Vierfingerfurche und dysplastischer Ohrmuscheln mit Ohranhängseln festzustellen.

#### Einschätzung und Therapie.

Die Diagnosen einer ADHS und eines Tourette-Syndroms wurden bestätigt. Zusätzlich wurde eine gemischte Zwangsstörung diagnostiziert. Die zuletzt als wirksam und verträglich eingeschätzte Kombinationstherapie mit Pimozid und Aripiprazol wurde erneut eingeleitet und mit Sertralin augmentiert. Hierunter zeigte sich eine stabile Teilremission des Beschwerdebildes. Zusätzlich wurde die Einleitung einer ergotherapeutischen und verhaltenstherapeutischen Behandlung mit „Habit-reversal“-Elementen empfohlen. Der Patient zeigte v. a. Auffälligkeiten in den Subkonstrukten der RDoC-Domänen „kognitive Systeme“ und „sensomotorische Systeme“. Interessanterweise zeigte sich eine Überschneidung mit Patientin A bei Auffälligkeiten innerhalb des Subkonstrukts „Arbeitsgedächtnis“ der Domäne „kognitive Prozesse“.

## Diskussion

Die vorgestellten Fälle zeigen exemplarisch die Heterogenität der klinisch-neuropsychiatrischen Manifestationen von 22q11.2-Mikrodeletionssyndromen. Die psychosozialen Beeinträchtigungen beider Patient*innen waren trotz wiederholter Behandlungsversuche erheblich, das Funktionsniveau überdauernd reduziert. Eine konsequente Erfassung der Merkmale dieser Patientin*innen im Sinne der RDoC-Analyseeinheiten, wie Tab. 1 sie für ausgewählte RDoC-Domänen vorschlägt, könnte die frühe Anwendung passgenauer Behandlungsstrategien erleichtern. Hier zeigten sich Überlappungen zwischen beiden Patient*innen im Besonderen des Subkonstrukts „Arbeitsgedächtnis“ der RDoC-Domäne „kognitive Prozesse“, die alleine anhand des klinischen Eindrucks und unter Beachtung kategorialer diagnostischer Kriterien weder evident noch relevant gewesen wären. Spezifika von Funktionen des Arbeitsgedächtnisses stellen jedoch wahrscheinlich ein transdiagnostisch valides, genetisch assoziiertes und funktionell relevantes Merkmal psychischer Störungen dar, das eine optimierte Klassifikation und Stratifizierung von Patient*innen jenseits kategorialer Diagnosen ermöglichen könnte [21]. Die breite Anwendung der RDoC-Systematisierung im klinischen Alltag stellt also einen Weg zu einer transdiagnostischen Erfassung und Charakterisierung von Patient*innen im Sinne einer angewandten Neurowissenschaft in der Psychiatrie [22] dar. Dies ermöglicht gerade bei Patienten mit relativ umschriebenen genetischen Syndromen ein genaueres Mapping von neurobiologischen Korrelaten und spezifischen klinischen Merkmalen mit dem Ziel der Entwicklung personalisierter Therapien. Datengestützte Analysemethoden werden hier zunehmend von Bedeutung sein [7]. Im Umkehrschluss könnten hierdurch in exemplarischer Weise durch die Analyse von Merkmalsclustern bei überlappender genetischer Grundlage Rückschlüsse auf eine bessere genetische Binnendifferenzierung der Störungsbilder möglich werden. Während für andere Mikrodeletionssyndrome, z. B. das Williams-Beuren-Syndrom, die Zusammenhänge zwischen Verhaltensphänotyp, genetischen Merkmalen sowie Hirnfunktion und -architektur mittlerweile gut belegt [15, 16] sind, werden Patienten mit 22q11.2 vor allem im Hinblick auf kategoriale Krankheitskategorien untersucht [17]. Somit könnten Störungsbilder mit bekannter genetischer Grundlage und heterogener Psychopathologie, wie hier eindrucksvoll beschrieben, Modellcharakter im Hinblick darauf erlangen, wie durch konsequente Charakterisierung anhand von RDoC rasch ein fundierteres, differenzierteres Verständnis für die genetischen Grundlagen unterschiedlicher klinischer Manifestationen gewonnen werden kann [23].

**Table 1:** 

RDoC-Domäne	(Sub‑)Konstrukt	Skalen/Interviews	Testverfahren	Paradigmen	Klinische Manifestation/Symptome
Soziale Prozesse	Soziale Kommunikation	*SRS, ADOS‑2, MBAS*	–	–	*Prosodie, Sprachpramatik, Sprecherwechsel etc.*
	Wahrnehmung und Verständnis – Selbst	*AQ*	–	–	Kontrollüberzeugung
	Wahrnehmung und Verständnis – Andere	*EQ, AQ*	–	*RMET*	*Emotionserkennung*
Kognitive Systeme	Aufmerksamkeit	–	*TMT‑A, TMT‑B*	–	Ablenkbarkeit
	Wahrnehmung	–	WISC‑V	–	Wahrnehmungsgebundenes Lernen
	Sprache	–	WISC‑V	–	Verstehen, kohärente Sprachproduktion
	Deklaratives Gedächtnis	–	*MWT‑B*	*FWIT*	Lernen, Erinnerungsvermögen
	Kognitive Kontrolle	*WURS‑K*	–	–	Ablenkbarkeit, Impulsivität
	Arbeitsgedächtnis	–	*WMS‑R, *WISC‑V	–	**Planungs‑/Handlungskontrolle**
Sensomotorische Systeme	Motorik	YGTSS	–	–	*Stereotypien, *Tics
	Kontrollüberzeugung	YGTSS	–	–	Tics
	Erlerntes Verhalten	–	–	–	Zwänge
	Angeborene motorische Muster	–	–	–	*Stereotypien*

*ADOS‑2* Autism Diagnostik Observation Schedule Version 2, *AQ* Autism Quotient, *EQ* Empathy Quotient, *FWIT* Farbe-Wort-Interferenztest, *MBAS* Marburger Beurteilungsskala zum Asperger-Syndrom, *MWT‑B* Mehrfachwahl-Wortschatz-Intelligenztest Version B, *RMET* Reading the Mind in the Eye Test, *SRS* Social Responsiveness Scale, *WISC‑V* Wechsler Intelligence Scale for Children Version 5, *WMS‑R* Wechsler Memory Scale revidierte Fassung, *YGTSS* Yale Globale Tic-Schweregrad-Skala

^a^Patientin A: *kursiv*, Patient B: steil. Überschneidungen sind durch **Fettung** hervorgehoben

## Fazit für die Praxis

- Auch Störungen mit identischer oder sehr ähnlicher genetischer Grundlage können äußerst heterogene neuropsychiatrische Manifestationen aufweisen.
- 22q11.2-Mikrodeletionssyndrome könnten exemplarisch die Anwendbarkeit von RDoC im Hinblick auf eine genauere Binnendifferenzierung der Symptomatik komplexer Störungsbilder ermöglichen.
- Eine konsequente Charakterisierung dieser Patient*innen anhand der RDoC-Analyseeinheiten im klinischen Alltag im Sinne einer angewandten Neurowissenschaft in der Psychiatrie erscheint sinnvoll, um langfristig passgenaue Behandlungsstrategien zu entwickeln.
- Über kategoriale Klassifikationen hinaus können somit transdiagnostische Erkenntnisse und Behandlungsmethoden rasch Eingang in die klinische Praxis finden.
